# Sigmoid Diverticulitis in a Tight Spot: An Atypical Presentation Within a Ventral Hernia

**DOI:** 10.7759/cureus.50875

**Published:** 2023-12-20

**Authors:** Constantine Ezeme, Grace Amaefule-Orie, Trevor M Yeung, Richard Bowyer

**Affiliations:** 1 Department of Surgery, Sheffield Teaching Hospitals, NHS Foundation Trust, Sheffield, GBR; 2 Department of Surgery, St. Richard's Hospital, Chichester, GBR; 3 Department of Anesthesia, Northampton General Hospital NHS Trust, Northampton, GBR; 4 Department of Colorectal Surgery, Memorial Sloan Kettering Cancer Center, New York City, USA

**Keywords:** case report, emergency surgery, stoma, ventral hernia, diverticulitis

## Abstract

Ventral hernia and acute diverticulitis may present with similar symptoms posing difficulty in clinical diagnosis. Rarely, complicated sigmoid diverticulitis is found within an irreducible ventral hernia sac in the emergency setting. Intraoperative decision on the appropriate surgical option depends on the surgeon’s experience and the patient’s clinical state.

We present a case of a middle-aged female who came in with infraumbilical hernia containing necrotic sigmoid diverticulitis. Her surgical history was cesarean section and total abdominal hysterectomy with a re-look laparotomy. She had an emergency exploration of the hernia through a midline incision, excision of the necrotic diverticulum, and the formation of loop colostomy at the site of the hernia. Post-operative recovery was uneventful and she has been scheduled for an elective sigmoid colectomy and reversal of the stoma. This study highlights that complicated sigmoid diverticulitis can rarely present as an irreducible ventral hernia and that less is often more in safely getting patients out of trouble in an emergency.

## Introduction

Acute diverticulitis and complicated hernia are common reasons for accident and emergency visits and in some instances the presenting symptoms may be similar [1-4]. However, the finding of complicated acute diverticulitis in a ventral hernia is rare [5]. The diagnosis of complicated hernia is mainly clinical, and depending on the clinical features at presentation, a patient may be resuscitated and taken to the theatre for surgery without cross-sectional imaging. The question of the optimum surgical approach, when complicated diverticulitis is found in a ventral hernia sac in the emergency setting, remains unanswered. In this study, we present a safe surgical option and review the literature on the surgical management of complicated diverticulitis in a hernia sac.

## Case presentation

**Figure 1:**
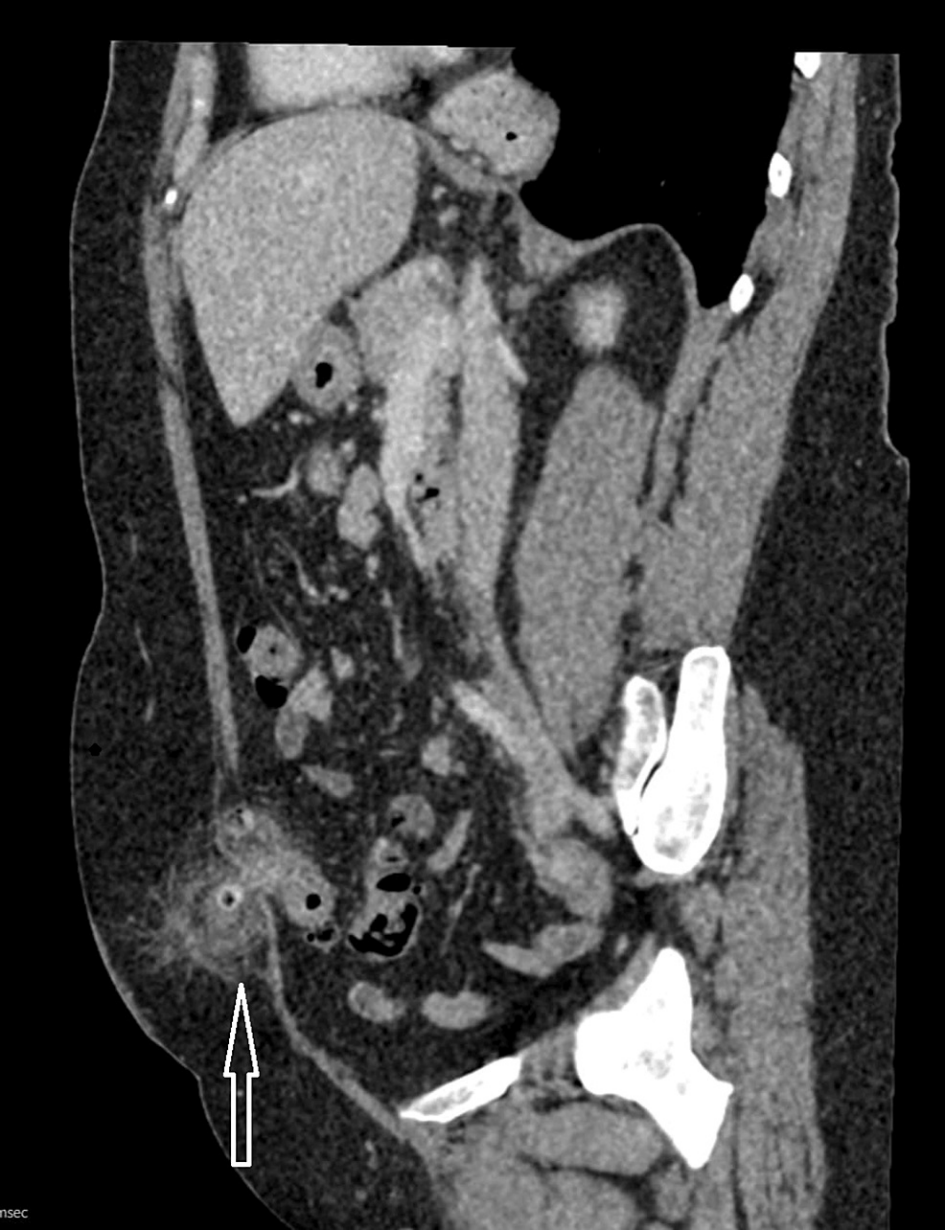
Sagittal view CT scan of the abdomen showing the ventral hernia containing sigmoid diverticulitis.

**Figure 2:**
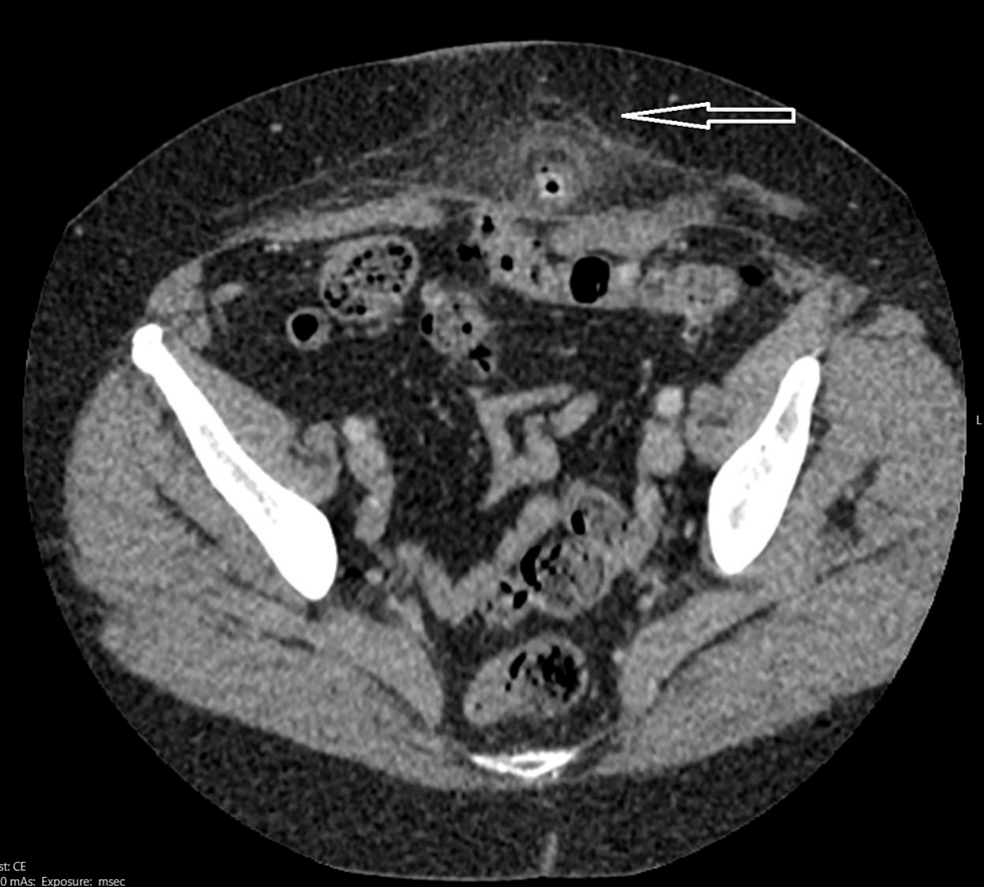
Axial view CT scan of the abdomen showing ventral hernia sac containing sigmoid diverticulitis.

A 55-year-old lady presented with a three-day history of lower abdominal pain worse at the site of an infraumbilical lump. There was nausea, abdominal distension, and constipation but no vomiting. She was an ex-smoker with a BMI of 27.5 kg/m^2^ who had a cesarean section and total abdominal hysterectomy with re-look laparotomy for postoperative hemorrhage, over a decade ago. Her vital signs were normal. On examination, she had a 4x4 cm infraumbilical mass with overlying erythema that was separate and superior to her Pfannenstiel incision. It was tense, tender, irreducible, and had no cough impulse but there were normal bowel sounds. Blood tests showed a normal lactate but raised inflammatory markers. Her abdominal CT scan was reported as showing a midline infraumbilical ventral hernia containing an inflamed sigmoid diverticulum (Figures 1, 2).

**Figure 3:**
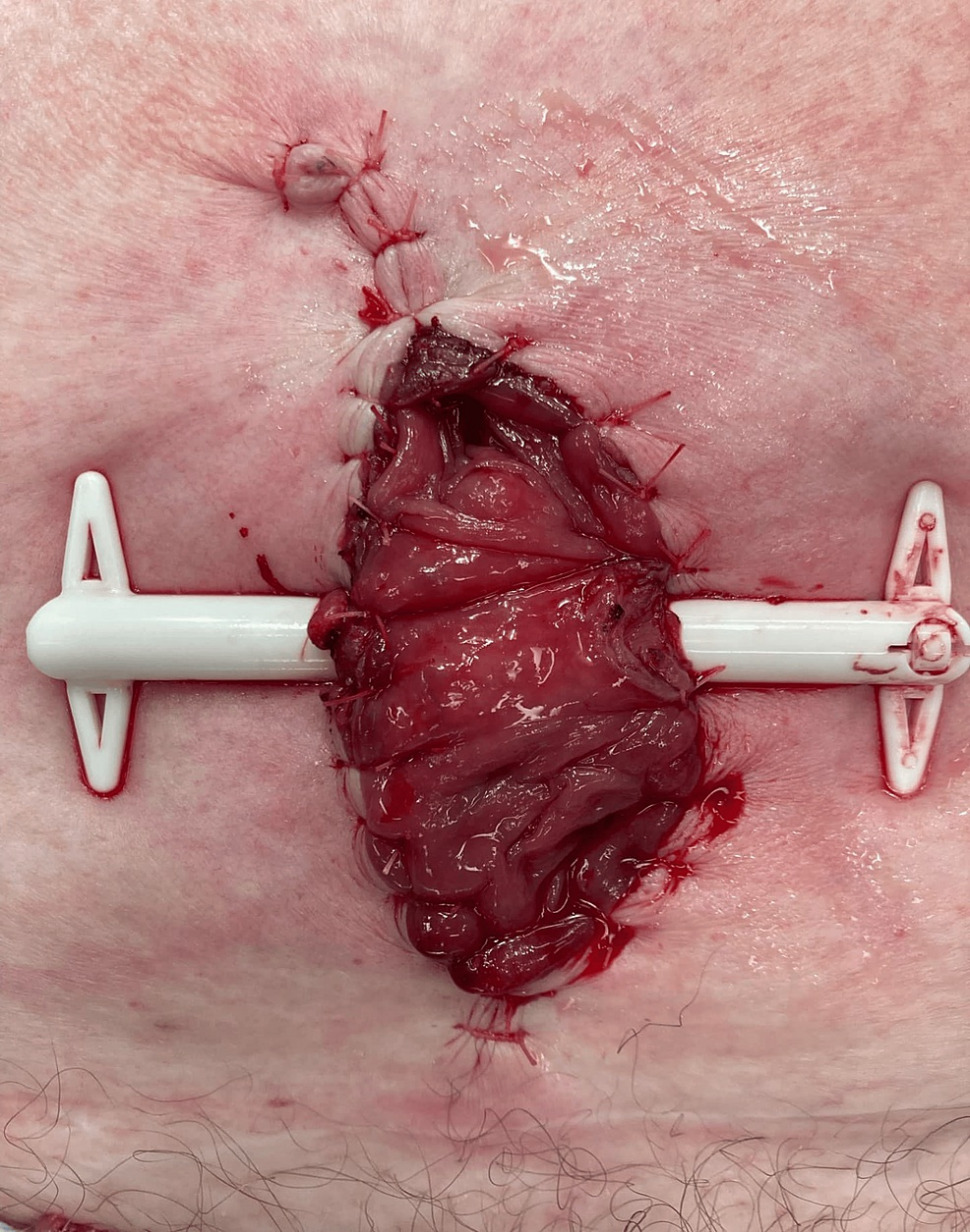
Loop sigmoid colostomy fashioned at the site of the ventral hernia.

She underwent an emergency exploration of the hernia through a vertical midline incision. The hernia sac was found to contain a necrotic sigmoid diverticulum which was not perforated. The necrotic diverticulum was excised, and loop colostomy was fashioned at the hernia site (Figure 3). She made a good recovery and was discharged on postoperative day nine. She is scheduled for an elective sigmoid colectomy and restoration of bowel continuity in due course.

## Discussion

Ventral hernia and acute diverticulitis may present with similar symptoms causing diagnostic difficulties [1-4]. There has been a report of Bochdalek hernia with incarcerated splenic flexure of the colon [6], and two reports of spigelian hernia mimicking diverticulitis [1,2]. Although uncommon, there are more reports of diverticulitis presenting as hernias. We found eight cases of diverticular abscess manifesting as a complicated groin hernia [3,4,7-10], and 10 reports of diverticulitis being a content of groin hernia sac. Among these cases, one was cecal diverticulitis [11], another was transverse colon diverticulitis [5], and the remaining ones involved the sigmoid colon [12-19].

We present a highly unusual case of necrotic sigmoid diverticulitis in a strangulated midline ventral hernia which has not been reported in the literature. The clinical features at presentation suggest a possible obstructed infraumbilical hernia but given the duration of the symptoms and raised inflammatory markers, a CT scan of the abdomen was done to better define the pathology and plan for surgery. The operative approach in the case was a midline incision at the site of the hernia, through which the necrotic diverticulum was excised, and a loop sigmoid colostomy fashioned at the site of the incision.

Clinical assessment may be sufficient to make a diagnosis and plan for surgery in many complicated hernias in the emergency setting but when there are diagnostic difficulties or suspicion of an alternate diagnosis, a preoperative CT scan may be beneficial in obtaining informed consent from the patient and planning for surgical intervention. In the reported cases of diverticular abscess mimicking complicated groin hernia, preoperative imaging aided diagnosis and planning for surgery in four of the eight cases. Surgical access was gained through a midline incision in these cases which guaranteed better exposure and the completion of the operation through a single incision in three of them [4,8], but one had a groin incision which was abandoned for a midline laparotomy [4]. In four other cases, the patients were taken to the theatre on grounds of clinical diagnosis, three had groin exploration which was abandoned for a midline incision [10] or used for drainage of abscess where the patients had CT scan in the early postoperative period for diagnosis and definitive surgical intervention [7,9]. The fourth case was intended for a laparoscopic repair which was abandoned on the finding of extensive adhesion at the internal inguinal ring. CT scan done after the attempted laparoscopy repair confirmed an abscess at the groin from sigmoid diverticulitis [3]. The reports demonstrate the benefit of preoperative imaging in an unusual presentation of common surgical conditions.

The optimum surgical intervention for the management of complicated diverticulitis (necrotic, perforated, or an abscess) found in an incarcerated hernia is debatable and depends on the clinical status of the patient and the choice of the surgeon. We performed a wedge resection of the necrotic diverticulum and fashioned a loop stoma at the incision site in this case. This was the least invasive approach and likely to elicit the least response to surgical trauma in this scenario. It was therefore considered as the safest option for the patient at the time of the surgery.

Petersen and Valentino suggested that the first surgical option for a hernia containing complicated diverticulitis is the resection of the inflamed colon and formation of a stoma at the site of the hernia, followed by restoration of the bowel continuity later [15]. This was the approach used by Kouraklis et al. for an incarcerated groin hernia containing a perforated single diverticulum and it is the principle applied in our case [19]. The second option is Hartmann’s procedure through a midline laparotomy [13,14,18], and in cases where conversion from groin to midline incision, a Bassini repair of the groin incision may be performed [13]. A third approach is the resection of the involved colon with primary anastomosis. This has been reported in a case of a single perforated diverticulum with a stoolball in an otherwise not inflamed sigmoid colon [15] and in the setting of perforated diverticulitis with localized peritonitis [12]. The outcome in each of these two scenarios was satisfactory, but primary anastomosis in the presence of inflammation has a high risk of leakage and may not be a safe option.

Patient perspective

She felt very anxious about the diagnosis of a trapped inflamed segment of the large bowel in the hernia which required urgent operation but was reassured. In her words “The care I received while in the hospital was exemplary on all levels. The consultant took time to explain everything, and I trusted him.” Finding she had a stoma after surgery was devastating, although she had been warned of the possibility. She reported that her admission into a ward with side rooms afforded her the privacy to adapt to the new situation. In her words “The stoma nurses were quite creative due to the difficult position of the stoma. Psychologically it was a very difficult time and I’ll admit to a few tears. I’m a single parent and we are dependent on my income. It was all very sudden and what should have been a normal working day ended with me in the theatre with extensive time off work.”

## Conclusions

In conclusion, this study highlights the importance of preoperative imaging when there are diagnostic difficulties and in situations of unusual presentation of common surgical emergencies. Stoma formation at the site of the ventral hernia is a simple and safe surgical option in the management of necrotic acute diverticulitis in a ventral hernia. It underscores the fact that the simplest procedure may often be the best option for the patients in safely getting them out of trouble in an emergency with definitive surgery planned later.
